# Cost-effective external interference for promoting the evolution of cooperation

**DOI:** 10.1038/s41598-018-34435-2

**Published:** 2018-10-30

**Authors:** The Anh Han, Long Tran-Thanh

**Affiliations:** 1 School of Computing, Media and the Arts, Teesside University, Borough Road, Middlesbrough, TS1 3BA UK; 2 School of Electronics and Computer Science, University of Southampton, University Road, Southampton, SO17 1BJ UK

## Abstract

The problem of promoting the evolution of cooperative behaviour within populations of self-regarding individuals has been intensively investigated across diverse fields of behavioural, social and computational sciences. In most studies, cooperation is assumed to emerge from the combined actions of participating individuals within the populations, without taking into account the possibility of external interference and how it can be performed in a cost-efficient way. Here, we bridge this gap by studying a cost-efficient interference model based on evolutionary game theory, where an exogenous decision-maker aims to ensure high levels of cooperation from a population of individuals playing the one-shot Prisoner’s Dilemma, at a minimal cost. We derive analytical conditions for which an interference scheme or strategy can guarantee a given level of cooperation while at the same time minimising the total cost of investment (for rewarding cooperative behaviours), and show that the results are highly sensitive to the intensity of selection by interference. Interestingly, we show that a simple class of interference that makes investment decisions based on the population composition can lead to significantly more cost-efficient outcomes than standard institutional incentive strategies, especially in the case of weak selection.

## Introduction

The study of the evolution of cooperation in populations of self-interested individuals has been given significant attention in a number of disciplines, ranging from Evolutionary Biology, Economics, Physics, Computer Science and Social Science. Various mechanisms that can promote the emergence and stability of cooperative behaviours among such individuals, have been proposed^1–4^. They include kin and group selection^5,6^, direct and indirect reciprocities^7–11^, spatial networks^12–19^, reward and punishment^20–25^, and pre-commitments^26–30^. In these works, the evolution of cooperation is typically originated from the emergence and stability of participating individuals’ strategic behaviours, which are cooperative in nature (e.g. direct reciprocity interactions are dominated by reciprocal strategies such as tit-for-tat like strategies, who tend to cooperate with alike individuals, leading to end populations with high levels of cooperation^8^). In other words, these mechanisms are incorporated as part of individual strategic behaviours, in order to study how they evolve in presence of other possible behaviours and whether that leads to a better outcome for cooperative behaviour.

However, in many scenarios, cooperation promotion or advocation is carried out by an *external* decision-maker or agent (i.e. the agent does not belong to the system). For instance, international institutions such as the United Nations and the European Union, are not inside parties to any nation’s political life (and thus, can be considered as an outsider from that nation political parties’ perspective), might want to promote a certain preferred political behaviour^31^. To do so, the organisation can provide financial support to political parties that choose to follow the preferred politics. Another example is wildlife management organisations (e.g., the WWF) aiming to maintain a desired level of biodiversity of a certain region. To do so, the organisation, which is not part of the region’s eco-system, has to decide whether to modify the current population of some species, and if so, then when, and to what degree she is required to *interfere* in the eco-system (i.e., modify the size and the biodiversity of the population)^32,33^. Since a more efficient population controlling typically implies more physical actions, thereby requiring higher (monetary) expenses in both human resources and equipments, the organisation has to balance between an efficient management and a low total investment cost. Moreover, under evolutionary dynamics of an eco-system, consisting of various stochastic effects such as those resulting from behavioural update and mutation, undesired behaviours can reoccur over time, if the interference was not sufficiently or efficiently carried out in the past. Given this, the external decision-maker also has to take into account the fact that she will have to repeatedly interfere in the eco-system, in order to sustain the level of the desired behaviour over time. That is, she has to find an efficient iterative interference scheme that leads to her desired goals, while minimising the total cost of interference.

Herein we study how to promote the evolution of cooperation within a well-mixed population or system of self-regarding individuals or players, from the perspective of external decision-makers. The individuals’ interaction is modelled using the one-shot (i.e. non-repeated) Prisoner’s Dilemma, where defection is always preferred to cooperation^1,6,7,12^. Suppose that the external decision-maker has a budget to use to intervene in the system by rewarding particular individuals in the population at specific moments. Such a decision is conditional on the current behavioural composition of individuals within the population, where at each time step or generation, those exhibiting a cooperative tendency are to be rewarded from the budget. However, the defective (i.e. non-cooperative) behaviour can reoccur over time through a mutation or exploration process^34^ and become prevalent in the population; thus, the decision-maker has to repeatedly interfere in the system in order to maintain the desired abundance of cooperators in the long run.

The research question here is to determine when to make an investment (i.e., pay or reward a cooperative act) at each time step, and by how much, in order to achieve our desired ratio of cooperation within the population such that the total cost of interference is minimised. We formalise this general problem of cost-efficient interference as a bi-objective optimisation problem, where the first objective is to provide a sequential interference scheme (i.e., a sequence of interference decisions over time) that maximises the frequency of cooperative behaviours within the population, while the second is to minimise the expected total cost of interference.

We will describe general conditions ensuring that an interference scheme can achieve a certain level of cooperation. Moreover, given a budget, we investigate how spreading the interference scheme should be to achieve a desired level of cooperation at a minimal cost. In other words, we will identify under what condition of the population composition should one stop making costly interference without affecting the desired cooperative outcome? To this end, we will develop an individual-based investment scheme that generalises the standard models of institutional incentives (i.e. institutional reward and punishment)^35–38^ where incentives are provided conditionally on the composition of the population. Our analysis shows that this individual-based investment strategy is more cost-effective than the standard models of institutional incentives for a wide range of parameter values.

## Results

### Interference scheme in finite populations

Let us consider a well-mixed, finite population of *N* self-regarding players, who interact with each other using the one-shot Prisoner’s Dilemma (PD), where a player can be either a cooperator (C) or defector (D) strategy. The payoff matrix of the PD is given as follows

$$\begin{array}{c}C\\ D\end{array}(\begin{array}{ll}C & D\\ R,R & S,T\\ T,S & P,P\end{array}).$$

That is, if both players choose to play C or D, they both receive the same reward *R* for mutual cooperation or penalty *P* for mutual defection. For the unilateral cooperation case, the cooperative player receives the payoff *S* and the defective one receives payoff *T*. The payoff matrix corresponds to the preferences associated with the PD when the parameters satisfy the ordering *T* > *R* > *P* > *S*. Extensive theoretical analysis has shown that for cooperation to evolve in a PD certain mechanisms such as repetition of interactions, reputation effects, kin and group relations, structured populations, or pre-commitments, need to be introduced^2^ (cf. Introduction). Differently, in the current study we focus on how an external decision-maker can interfere in such a population of C and D players to achieve high levels of cooperation in a cost-efficient way.

Indeed, with a limited budget, we analyse what would be the optimal interference or investment scheme that leads to the highest possible frequency of cooperation. In the current well-mixed population setting, an interference scheme solely depends on the current composition of the population, i.e., whenever the population consists of *i* C players (and thus, *N* − *i* D players), a total investment, *θ*_*i*_, is made. That is, each C player’s average payoff is increased by an amount *θ*_*i*_/*i*. Let Θ = {*θ*_1_, ...., *θ*_*N*−1_} be the overall interference scheme. Our goal is thus to find Θ that minimises the expected (total) cost of interference while maximising or at least ensuring a certain level of cooperation.

Besides providing the analysis for such a general interference scheme, we will consider an *individual-based investment scheme* where there is a fixed investment per C-player, i.e. *θ*_*i*_ = *i* × *θ*. This individual-based merit or rewarding is widespread, as are the cases for scholarships and performance based payments^39^. Within this scheme, we investigate whether one should spend the budget on a small number of *C* players rather than spreading it to pay all C players though that might not be sufficient for them to survive (especially when the resource is limited). That is, a C player might not be competitive or strong enough to survive when receiving a too small investment, leading to the waste of such an investment. To do so, we consider investment schemes that reward C players whenever their frequency or number in the population does not exceed a given threshold *t*, where 1 ≤ *t* ≤ *N* − 1. Hence, we have ${\theta }_{k}=k\times \theta \,\forall k\le t$ and =0 otherwise.

It is noteworthy that this interference scheme generalises incentive strategies typically considered in thse literature of institutional incentives modelling, i.e. institutional reward and punishment^36–38^, where incentives are always provided regardless of the population composition. That is, those works only consider the most extreme case of the individual-based incentive scheme where *t* = *N* − 1 (denoted by FULL-INVEST). Our analysis below shows that in most cases there is a wide range of *t* that leads to a lower total cost of investment than FULL-INVEST while guaranteeing the same cooperation outcome.

### Cost-effective interference that ensures cooperation

We now derive the expected cost of interference with respect to the interference scheme Θ. We adopt here the finite population dynamics with the Fermi strategy update rule^40^ (see Methods), stating the probability that a player *A* with fitness *f*_*A*_ adopts the strategy of another player *B* with fitness *f*_*B*_ is given by the Fermi function, i.e ${P}_{A,B}={(1+{e}^{-\beta ({f}_{B}-{f}_{A})})}^{-1}$, where *β* stands for the selection intensity.

We now derive the formula for the expected number of times that the population consists of *i* C players. To that end, let us denote by *S*_*i*_, 0 ≤ *i* ≤ *N*, the state in which the population consists of *i* C players (and thus, *N*−*i* D players). These (*N* + 1) states define an absorbing Markov chain, with *S*_0_ and *S*_*N*_ being the absorbing states. Let $U={\{{u}_{ij}\}}_{i,j=1}^{N-1}$ be the transition matrix between the transient states, i.e. {*S*_1_, ..., *S*_*N*−1_}. Clearly, *U* forms a tridiagonal matrix (i.e. ${u}_{k,k\pm j}=0\,\forall j\ge 2$), with the elements on the upper and lower diagonals being defined by the probabilities that the number of C players (*k*) in the population is increased or decreased by 1, respectively. These probabilities are denoted by *T*^±^(*k*) (see Methods); thus, *u*_*k*,*k*±1_ = *T*^±^(*k*). Finally, the elements on the main diagonal of *U* are defined as, ${u}_{k,k}=1-{u}_{k,k+1}-{u}_{k,k-1}=1-{T}^{+}(k)-{T}^{-}(k)$.

As such, we can form the (so-called) fundamental matrix $N={\{{n}_{ij}\}}_{i,j=1}^{N-1}={(I-U)}^{-1}$, where *n*_*ij*_ defines the expected number of times the population spends in the state *S*_*j*_ given that it starts from (non-absorbing) state *S*_*i*_^41^. Thus, assuming that a mutant can occur, with equal probability, at either of the homogeneous population states where the population consists of only C or D players (i.e. *S*_0_ or *S*_*N*_), the expected number of visits at state *S*_*i*_ is given by: (*n*_1*i*_ + *n*_*N*−1,*i*_)/2. Therefore, the expected interference or investment cost for the investment scheme Θ = {*θ*_1_, ...., *θ*_*N*−1_} is given by,

1 $$EC=\frac{1}{2}\sum _{i=1}^{N-1}({n}_{1i}+{n}_{N-\mathrm{1,}i}){\theta }_{i}.$$

We now calculate the frequency (or fraction) of cooperation when the interference scheme Θ = {*θ*_1_, ...., *θ*_*N*−1_} is applied. Since the population consists of only two strategies, the fixation probabilities of a C (respectively, D) player in a (homogeneous) population of D (respectively, C) players when the interference scheme is carried out are (see Methods), respectively,

2 $$\begin{array}{rcl}{\rho }_{D,C} & = & {(1+\sum _{i=1}^{N-1}\prod _{k=1}^{i}\frac{1+{e}^{\beta ({{\rm{\Pi }}}_{k}(C)-{{\rm{\Pi }}}_{k}(D)+{\theta }_{k}/k)}}{1+{e}^{-\beta ({{\rm{\Pi }}}_{k}(C)-{{\rm{\Pi }}}_{k}(D)+{\theta }_{k}/k)}})}^{-1},\\ {\rho }_{C,D} & = & {(1+\sum _{i=1}^{N-1}\prod _{k=1}^{i}\frac{1+{e}^{\beta ({{\rm{\Pi }}}_{k}(D)-{{\rm{\Pi }}}_{k}(C)-{\theta }_{k}/k)}}{1+{e}^{-\beta ({{\rm{\Pi }}}_{k}(D)-{{\rm{\Pi }}}_{k}(C)-{\theta }_{k}/k)}})}^{-1}.\end{array}$$

Computing the stationary distribution using these fixation probabilities, we obtain the frequency of cooperation (see Methods)

$$\frac{{\rho }_{D,C}}{{\rho }_{D,C}+{\rho }_{C,D}}.$$

Hence, this frequency of cooperation can be maximised by maximising

3 $$\mathop{{\rm{\max }}}\limits_{{\rm{\Theta }}}({\rho }_{D,C}/{\rho }_{C,D}).$$

The fraction in Equation (3) can be simplified as follows^34^

4 $$\begin{array}{rcl}\frac{{\rho }_{D,C}}{{\rho }_{C,D}} & = & \prod _{k=1}^{N-1}\frac{{T}^{-}(k)}{{T}^{+}(k)}=\prod _{k=1}^{N-1}\frac{1+{e}^{\beta [{{\rm{\Pi }}}_{k}(C)-{{\rm{\Pi }}}_{k}(D)+{\theta }_{k}/k]}}{1+{e}^{-\beta [{{\rm{\Pi }}}_{k}(C)-{{\rm{\Pi }}}_{k}(D)+{\theta }_{k}/k]}}\\  & = & {e}^{\beta \sum _{k=1}^{N-1}({{\rm{\Pi }}}_{k}(C)-{{\rm{\Pi }}}_{k}(D)+{\theta }_{k}/k)}\\  & = & {e}^{\beta (N(R+S-T-P)/2+(P-R)+\sum _{k=1}^{N-1}{\theta }_{k}/k)}\mathrm{.}\end{array}$$

In the above transformation, *T*^−^(*k*) and *T* ^+^(*k*) are the probabilities to increase or decrease the number of C players (i.e. *k*) by one in each time step, respectively (see Methods for details). Since the payoff matrix entries of the PD (i.e. *R*, *T*, *P*, *S*) are fixed, in order to guarantee a certain level of cooperation, we only need to examine the following quantity (which increases with Θ)

5 $$G=\sum _{k=1}^{N-1}{\theta }_{k}/k.$$

In short, the described optimisation problem is reduced to the problem of finding an interference scheme, Θ = {*θ*_1_, ...., *θ*_*N*−1_}, that maximises the level of cooperation within the population, by maximising *G*, while minimising the expected interference cost *EC*, as defined in Equation (1).

### Sufficient conditions for achieving cooperation by the overall scheme

We now derive conditions for the overall interference scheme Θ that can ensure a certain level of cooperation. In particular, from Equation (4), we can derive that cooperation has larger basin of attraction than that of defection (i.e., *ρ*_*D*,*C*_ > *ρ*_*C*,*D*_)^2^ if and only if

$$G\ge N(T+P-R-S)/2+(R-P).$$

In this case, this condition also means there will be at least 50% of cooperation. There is exactly 50% cooperation when *β* = 0, i.e. under neutral selection, regardless of the interference scheme in place. It implies that under neutral selection, it is optimal to make no investment at all, i.e. *θ*_*i*_ = 0 for all 1 ≤ *i* ≤ *N* − 1.

We henceforth only consider non-neutral selection, i.e. *β* > 0. Generally, assuming that we desire to obtain at least an *ω* ∈ [0, 1] fraction of cooperation, i.e. $\frac{{\rho }_{D,C}}{{\rho }_{D,C}+{\rho }_{C,D}}\ge \omega$, it follows from Equation (4) that

6 $$G\ge \frac{1}{\beta }\,\mathrm{log}(\frac{\omega }{1-\omega })+N(T+P-R-S)/2+(R-P).$$

Therefore it is guaranteed that if Θ satisfies this inequality, at least an *ω* fraction of cooperation can be expected. From this condition it implies that the lower bound of *G* monotonically depends on *β*. Namely, when *ω* ≤ 0.5, it increases with *β* while decreases for *ω* < 0.5. Note that *G* is an increasing function of the overall interference cost (vector) Θ.

### Sufficient conditions for individual-based scheme

We now apply this general condition to the individual-based investment scheme defined above. Recall that in this case, ${\theta }_{k}=k\times \theta \,\forall k\le t$ and =0 otherwise. Thus, *G* = *t* × *θ*. Hence, to obtain at least *ω* fraction of cooperation the per-individual investment cost *θ* needs to satisfy that

7 $$\theta \ge \frac{1}{t}(\frac{1}{\beta }\,\mathrm{log}(\frac{\omega }{1-\omega })+N(T+P-R-S)/2+(R-P)).$$

On the other hand, the threshold *t* must satisfy that

8 $$t\ge \frac{1}{\theta }(\frac{1}{\beta }\,\mathrm{log}(\frac{\omega }{1-\omega })+N(T+P-R-S\mathrm{)/2}+(R-P)).$$

These conditions suggest that, when *ω* ≥ 0.5, the smaller the intensity of selection (*β*) is, the larger the threshold of the per-individual investment (*θ*) as well as the higher the threshold for how spreading the investment must be (*t*) are required to achieve a given *ω* fraction of cooperation. It is reversed for *ω* < 0.5.

### Intermediate *t* leads to cost-effective investment strategies

**Figure 1:**
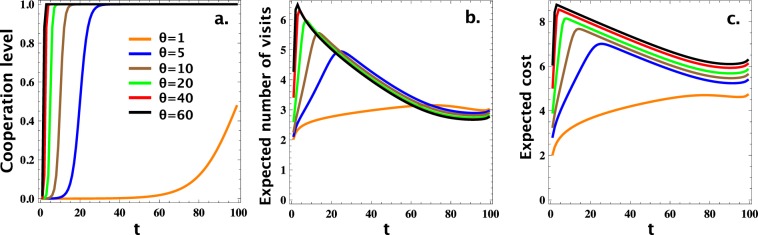
Level of cooperation (panel a), expected number of interferences (panel b), and expected total cost of interference (panel c), all as a function of the interference threshold *t* and for different values of *θ*. In panel (b) and (c), the results are scaled by Log(10). Parameters: *R* = 1, *T* = 2, *P* = 0, *S* = −1; *N* = 100; *β* = 0.1.

We now provide numerical simulation results for the individual-based investment scheme, computing the resulting stationary distribution in a population consisting of the two strategies, C and D (see Methods). Namely, Fig. 1a shows the level of cooperation as a function of the threshold *t*, for different values of the cost, *θ*. As expected, the level of cooperation obtained increases when *t* or *θ* increases. When *θ* is too small (see *θ* = 1), defection is prevalent, namely, it is always more frequent than cooperation, even when the investment is always made (i.e. *t* = *N* − 1). When this investment cost is large enough, a high level of cooperation can be sustained with a large *t*, i.e. when the investment is sufficiently spreading. The results are in accordance with the theoretical results in Eqs (7) and (8). For instance, with *θ* = 1, to reach at least *ω* = 0.4 (fraction of cooperation), it must satisfy that *t* ≥ 97. To reach at least *ω* = 0.5, it must satisfy that *t* ≥ 101, which means it is not possible to reach this level of cooperation given the cost. On the other hand, with sufficiently high values of the cost, one can reach significant cooperation with a rather small threshold for *t*. For example, with *θ* = 5 and 40, one can reach 99% of cooperation (i.e. *ω* = 0.99) whenever *t* ≥ 30 and *t* ≥ 4, respectively.

Thus, a question arises as to whether it is always the case that a more spreading interference scheme requires a larger budget (EC)? In other words, should we simply use the smallest possible value of *t* that leads to the required level of cooperation? When stochastic factors, such as mutation and frequency-dependence dynamics, are absent, clearly that is the case. However, when such stochastic factors are present, the answer is not obvious anymore. Indeed, as shown in Fig. 1c, when *t* reaches a threshold, a more spreading investment scheme can actually lead to decrease in the total investment. This observation can be explained by looking at the expected number of times of investment (i.e. the total number of visits at states *S*_*i*_, *i* ≤ t), in Fig. 1b, for varying *t*. Even when the number of C-players in the population is rather large (i.e. large *t*), an investment might still be required as otherwise defection has a chance to resurface, thus wasting the earlier efforts and requiring further investments. However, when number of C players reaches a threshold (of approximately 90%), these C players can maintain their abundance by themselves, without requiring further investments.

**Figure 2:**
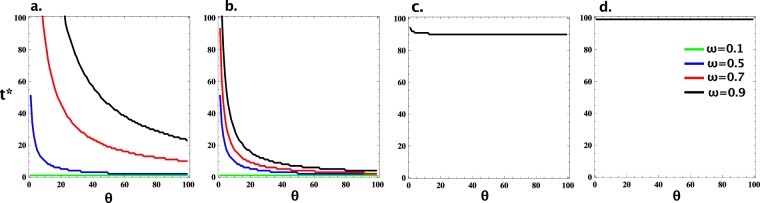
Optimal value t^*^ leading to an investment strategy with a minimal value of the expected cost of investment (EC), which guarantees at least *ω* frequency of cooperation. We study for varying individual cost of investment, *θ*, and for different intensities of selection, *β* (namely, *β* = 0.001, 0.01, 0.1, and 1, respectively, in panels a, b, c and d). In general, the value of t^*^ decreases with *θ* and increases with *ω* (comparing *ω* = 0.1, 0.5, 0.7 and 0.9). When *β* is sufficiently small (panels a, b and c), an intermediate value of t^*^ is always observed, while when *β* is sufficiently large (panel d), t^*^ must be the largest possible, i.e. t^*^ = *N* − 1. Parameters: *R* = 1, *T* = 2,*P* = 0, *S* = −1; *N* = 100.

Thus, as observed from results in Fig. 1, there is an intermediate value of *t* where an optimal (i.e. lowest) expected cost of investment is achieved. We denote this optimal value of *t* by t^*^. We now study how robust this observation is. Indeed, Fig. 2 shows t^*^ for varying *θ*, for different intensities of selection *β* and required levels of cooperation *ω*. In general, the value of t^*^ decreases with *θ* and increases with *ω* (comparing *ω* = 0.1, 0.5, 0.7 and 0.9). When *β* is sufficiently small (see panels for *β* = 0.001, 0.01, 0.1), an intermediate value of t^*^ is always observed, while when *β* is sufficiently large (see the panel for *β* = 1), t^*^ must be the largest possible, i.e. t^*^ = *N* − 1. That is, whenever selection is not too strong, we would expect to find the optimal interference scheme not always making an investment in cooperators, and the smaller the selection strength, the less spreading an investment scheme should be. As such, we might expect a wide range of *t* that leads to investment schemes that are more cost-effective than FULL-INVEST.

**Figure 3:**
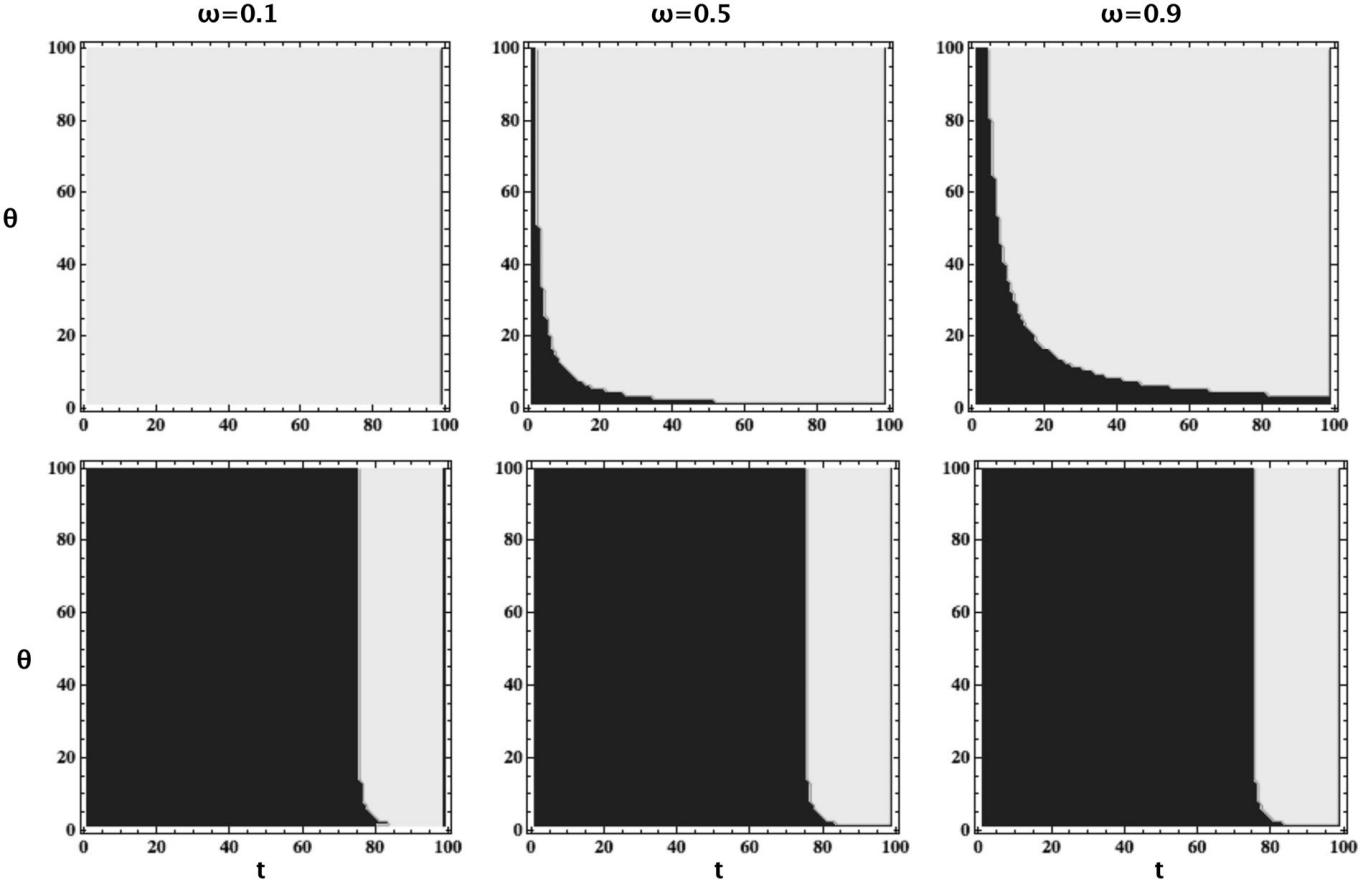
Range of *t* (grey area) that leads to investment schemes being more cost-efficient than FULL-INVEST (i.e. *t* = *N* − 1), guaranteeing at least *ω* fraction of cooperation, for varying per-individual investment cost *θ*. We plot for different values of *ω*: *ω* = 0.1 (left column), *ω* = 0.5 (middle column), *ω* = 0.9 (right column), and for different values of *β*: *β* = 0.01 (top row) and *β* = 0.1 (bottom row). In general, for a given *ω*, there is a large range of *t* leading to a more cost-efficient investment scheme than the FULL-INVEST. Parameters: *R* = 1, *T* = 2, *P* = 0, *S* = −1; *N* = 100.

Indeed, in Fig. 3, we study the range of *t* (grey area) that leads to investment schemes better than FULL-INVEST (i.e. *t* = *N* − 1), guaranteeing at least *ω* fraction of cooperation. We show that for varying *θ*, for different values of *ω* as well as intensities of selection *β*. In general, for a given required level of cooperation to be achieved, *ω*, there is a large range of *t* where it leads to a more cost-efficient strategy than the FULL-INVEST. This range is larger for a weaker intensity of selection *β*.

## Discussion

In summary, the present work seeks to answer the question of how to interfere in a population of self-interested players in order to promote high cooperation in a cost-effective way. In particular, the cost of interference is measured as the consumption of certain resources, and the higher impact we want to make, the higher cost we have to pay. To tackle this problem, we have developed a cost-efficient interference model based on evolutionary game methods in finite populations. In our model, an exogenous decision-maker aims to optimise the cost of interference while guaranteeing a certain minimum level of cooperation, having to decide whether and how much to invest (in rewarding cooperative behaviours) at each time step. For a general investment scheme, we have obtained the sufficient conditions for the scheme to achieve a concrete level of cooperation. Moreover, we have provided numerical analyses for a specific investment scheme that makes a fixed investment in a cooperator (i.e. individual-based investment) whenever the cooperation frequency in the population is below a threshold *t* (representing how widespread the investment should be to optimise the total cost of investment).

This individual-based scheme can be considered a more general form of the prevalent models of institutional incentive strategies, such as institutional punishment and reward^35–38,42–46^, which do not take into account the behavioural composition or state of the population. Typically, only the most extreme case is considered where incentives are always provided (punishment for defectors and reward for cooperators), which corresponds to *t* = *N* − 1 of the individual-based scheme. Our results have shown that whenever the intensity of selection is not too strong, an intermediate value of the threshold *t* leads to a minimal total cost of investment while guaranteeing at least a given desired fraction of population cooperation. Furthermore, there is a wide range of the threshold *t* where individual-based investment is more cost-effective than the above mentioned institutional incentive strategies; and the smaller the intensity of selection, the wider this range is.

Note that our work is also different from the existing institutional incentive models^35–38,42–46^, as well as the existing literature on the evolution of cooperation^1–4^, in that, its aim is to minimise the cost of interference while guaranteeing high levels of cooperation, while cost-efficiency is mostly ignored in those works. Similarly, our work also differs from EGT literature on optimal control in networked populations^47–49^, where cost-efficiency is not considered. Instead, these works on controllability focus on identifying which individuals or nodes are the most important to control (i.e. where individuals can be assigned strategies as control inputs), for different population structures.

Moreover, it is important to note that in the context of institutional incentives modelling, a crucial issue is the question of how to maintain the budget of incentives providing. The problem of who pays or contributes to the budget is a social dilemma itself, and how to escape this dilemma is critical research question. Facilitating solutions include pool incentives with second order punishments^42^, democratic decisions^37^, positive and negative incentives combination^36^ and spatial populations^44^, just to name a few. Our work does not address this issue of who to contribute to the budget, but rather focus on how to optimise the spending, given a budget already, which has not been addressed by these works. However, it would be interesting to study whether (and how) interference strategies should be customised for different types of incentive providers, which we aim to study in future work.

Furthermore, related to our work here is a large body of research on (sequential) decision-making in Artificial Intelligence and Multi-agent systems, which provide a number of techniques for making a sequence of decisions that lead to optimal behaviour of a system (e.g., a desired level of biodiversity), while minimising the total cost of making such decisions^50–54^. However, these lines of research often omit the agents’s intrinsic strategic behaviours, which clearly have a crucial role in driving the evolutionary dynamics and outcomes of agents’ interactions. Thus, these works failed to exploit the system intrinsic properties, and hence, not able to efficiently achieve a desired outcome and system status (e.g., the status quo between the fighting opponents, or the desired diversity of population).

On the other hand, game theoretic literature, which deals with agents’ intrinsic strategic behaviours, usually need to make simplistic assumptions. For instance, it is often the case that the system is assumed to be fully closed, having no external decision-makers; or, when there is an external decision-maker, he or she can fully control the system agents’ strategic behaviour. Examples of closed systems assumption are classical game theoretical models, see e.g. refs^52,55,56^. Approaches that require full control of agents’ behaviours are for example works from mechanism design, where the decision-maker is the system designer, who defines norms and penalties to ensure that agents are incentivised not to deviate from the desired behaviour, see e.g. refs^57–60^. Therefore, these works are not suitable to tackle our settings either.

Based on the general model we developed here, more efficient interference strategies can be studied. In particular, it would be interesting to consider more adaptive interference strategies, which modify the amount and the frequency of investment dynamically depending on the current state of the system. The analysis of the resulting systems, however, is not straightforward, as it remains unclear whether to increase or decrease the amount/frequency of investments will lead to more efficient performance. Moreover, we aim to extend our analysis to systems with other, more complicated scenarios such as structured populations and multi-player games, where more behavioural equilibria^61–63^ and structure-dependent^13,19^ interference strategies might be required to ensure cost-efficiency. In the former case, interference strategies would need to take into account the structural information in a network such as the cooperative properties in a neighbourhood (for the results of cost-efficient interference strategies in square lattice populations, see our recent work in ref.^64^). In the latter case, the strategies might need to consider the group size as well as cooperative properties in the group to decide whether to make an investment.

## Methods

Both the analytical and numerical results obtained here use Evolutionary Game Theory (EGT) methods for finite populations^34,65,66^. A similar description of the Methods section was used in refs^67,68^. In such a setting, players’ payoff represents their *fitness* or social *success*, and evolutionary dynamics is shaped by social learning^3,69^, whereby the most successful players will tend to be imitated more often by the other players. In the current work, social learning is modeled using the so-called pairwise comparison rule^40^, assuming that a player *A* with fitness *f*_*A*_ adopts the strategy of another player *B* with fitness *f*_*B*_ with probability given by the Fermi function, ${P}_{A,B}={(1+{e}^{-\beta ({f}_{B}-{f}_{A})})}^{-1}$, where *β* conveniently describes the selection intensity (*β* = 0 represents neutral drift while *β* → ∞ represents increasingly deterministic selection).

For convenience of numerical computations, but without affecting analytical results, we assume here small mutation limit^65,66,70^. As such, at most two strategies are present in the population simultaneously, and the behavioural dynamics can thus be described by a Markov Chain, where each state represents a homogeneous population and the transition probabilities between any two states are given by the fixation probability of a single mutant^65,66,70^. The resulting Markov Chain has a stationary distribution, which describes the average time the population spends in an end state.

Now, the average payoffs in a population of *k* A players and (*N* − *k*) B players can be given as below (recall that *N* is the population size), respectively,

9 $$\begin{array}{rcl}{{\rm{\Pi }}}_{A}(k) & = & \frac{(k-\mathrm{1)}{\pi }_{A,A}+(N-k){\pi }_{A,B}}{N-1},\\ {{\rm{\Pi }}}_{B}(k) & = & \frac{k{\pi }_{B,A}+(N-k-\mathrm{1)}{\pi }_{B,B}}{N-1}\mathrm{.}\end{array}$$

Thus, the fixation probability that a single mutant A taking over a whole population with (*N* − 1) B players is as follows (see e.g. references for details^40,65,71^)

10 $${\rho }_{B,A}={(1+\sum _{i=1}^{N-1}\prod _{j=1}^{i}\frac{{T}^{-}(j)}{{T}^{+}(j)})}^{-1},$$

where ${T}^{\pm }(k)=\frac{N-k}{N}\frac{k}{N}{[1+{e}^{\mp \beta [{{\rm{\Pi }}}_{A}(k)-{{\rm{\Pi }}}_{B}(k)]}]}^{-1}$ describes the probability to change the number of A players by ± one in a time step. Specifically, when *β* = 0, *ρ*_*B*,*A*_ = 1/*N*, representing the transition probability at neural limit.

Having obtained the fixation probabilities between any two states of a Markov chain, we can now describe its stationary distribution. Namely, considering a set of *s* strategies, {1, ..., *s*}, their stationary distribution is given by the normalised eigenvector associated with the eigenvalue 1 of the transposed of a matrix $M={\{{T}_{ij}\}}_{i,j=1}^{s}$, where ${T}_{ij,j\ne i}={\rho }_{ji}/(s-1)$ and ${T}_{ii}=1-{\sum }_{j=1,j\ne i}^{s}{T}_{ij}$. (See e.g. refs^66,70^ for further details)^72^.

## Data Availability

No datasets were generated or analysed during the current study.
